# A Systematic Review of Sildenafil Mortality Through the Years

**DOI:** 10.7759/cureus.32179

**Published:** 2022-12-04

**Authors:** Abdullah H Al Ibrahim, Khalid Q Ghallab, Fatima I Alhumaid, Husain H Almahfoudh, Ali J Almadan, Mohammed A Al Eid, Mujtaba H AlMishqab, Mohammed F Alsaffar, Jawad H Aljamea

**Affiliations:** 1 Medicine, Imam Abdulrahman Bin Faisal University, Al Khobar, SAU; 2 Pathology, Mouwasat Hospital Al Khobar, Al Khobar, SAU; 3 Medicine, Imam Abdulrahman Bin Faisal University, Alkhobar, SAU; 4 Medicine, Sarat Abidah General Hospital, Asir, SAU

**Keywords:** mortality, postmortem, death, viagra, phosphodiesterase-5 inhibitors, sildenafil

## Abstract

Sildenafil is a medication used for the treatment of erectile dysfunction. It was approved by the U.S. Food and Drug Administration (FDA) in 1998. Several articles have raised concerns regarding the use of sildenafil and the occurrence of serious adverse events, such as myocardial ischemia, stroke, and even death. Our aim is to systematically review the existing literature on mortality associated with sildenafil use. The method used for this systematic review was completed by searching three databases: PubMed, Scopus, and Web of Science. Articles were screened and assessed for eligibility. This review uses the articles found to address the concerns associated with sildenafil and mortality. A total of 19 reports were used in our systematic review, in which there were 10 case reports, two case series, three systematic reviews, one narrative review, one retrospective study, one article in the *British Medical Journal*, and one commentary article. One FDA article in particular included case reports and reports to the FDA on the use of sildenafil eight months after its introduction to the market in 1998, with 522 deaths reported. Another retrospective study examined the use of sildenafil on infants below the age of 1 who did not have congenital heart disease but did suffer from severe pulmonary hypertension. The study found a mortality rate of 29%, which increased with sildenafil dosage. A case series examined six deaths related to non-prescription use of sildenafil. All these cases were subjected to autopsies and related to sexual activity. The study suggests that phosphodiesterase 5 inhibitors induced the deaths, and the concentration of sildenafil in the femoral blood was found to be between 0.032and0.087 μg. To conclude, the literature available on this topic is deemed insufficient to provide enough data to establish a direct link of causality between sildenafil and mortality. Although some studies paint sildenafil as the culprit behind these deaths, further studies and research are needed to explain the unexpected deaths following sildenafil use.

## Introduction and background

Sildenafil, known by the brand name Viagra, is a medication that has been implicated in the management of a wide variety of common medical conditions. Primarily, it is prescribed in the form of a pill ranging from 25 mg (low dose ) to 100 mg (high dose) for the treatment of erectile dysfunction in males. Furthermore, it is approved to delay clinical worsening and improve exercise tolerance for patients with pulmonary hypertension. Other uses include the treatment of Raynaud’s phenomenon and female sexual arousal disorder and acting as an adjunct in the treatment of altitude-induced hypoxemia [1]. Sildenafil was patented in 1996 and approved by the U.S. Food and Drug Administration (FDA) on March 27, 1998, for treating erectile dysfunction, being the first oral medication for erectile dysfunction treatment in the United States [1]. In the last few years, sildenafil was one of the most prescribed drugs in the United States [2]. The introduction of sildenafil has made a valuable contribution to the treatment of erectile dysfunction by positively impacting the quality of life for patients suffering from anxiety, depression, or low self-esteem due to an impaired erection [1]. Sildenafil is an agent in a class of medications called phosphodiesterase (PDE) inhibitors, which work by competing with cyclic guanosine monophosphate (cGMP) to bind phosphodiesterase 5 (PDE5) in corpus cavernosum, maintaining higher levels of cGMP and causing the relaxation of blood-vessel smooth muscles, thereby improving blood inflow to the penis and enhancing penile erection. This results in a better response to sexual stimulation. Similar mechanisms result in vasodilation, which affects the peripheral vessels when used to treat Raynaud’s phenomenon and affects the pulmonary vessels when used to treat pulmonary hypertension. Sildenafil is metabolized in the liver by cytochrome P450 enzymes with a half-life of four hours. Cytochrome P450 is inhibited by a number of medications, resulting in higher plasma levels of sildenafil and an increased severity of side effects. Thus, attentive use of the drug by people on cytochrome P450 inhibitors, such as azithromycin, diltiazem, and colchicine, is advised [3]. Caution should be taken in patients with labile blood pressure readings as well due to the effect of the drug lasting up to 18 hours. Because sildenafil is a systemic vasodilator, patients with conditions resulting in blood pressure lability, such as left ventricular outflow obstruction and autonomic dysfunction, are at risk of hemodynamic instability. Patients taking medications with a synergetic effect, such as alpha-adrenergic blockers and other anti-hypertensive medications, are advised to initiate consumption of the drug with the lowest therapeutic dose. The co-administration of sildenafil and nitrate is contraindicated due to the risk of hemodynamic compromise. Commonly reported side effects include headaches, flushed skin, heartburn, dyspepsia, nasal congestion, back pain, and myalgia. Visual abnormalities, such as blue-green-tinged vision, increased perception of light, and blurred vision, have been reported in patients using sildenafil, especially at higher doses above 100 mg. Infrequently, sildenafil can result in a painful erection that is persistent for a prolonged period of time in absence of sexual stimulation, also known as priapism [1]. Hence, patients predisposed to priapism, such as those with sickle cell anemia or multiple myeloma, have to be alert when using the medication. Higher doses of sildenafil result in more side effects without increasing the drug’s efficacy [4]. There are some case reports that showed that sildenafil use might have electrophysiological effects, predisposing some patients to pro-arrhythmia and resulting in death [1,5-7]. A case report of sildenafil use described a man collapsing after sexual intercourse; he was saved from ventricular fibrillation by the Emergency Medical Services [8]. He had known coronary artery disease and took sildenafil without a prescription, leading to cardiac arrest. This case report prompted us to review sildenafil-associated mortality. In this systematic review, we aim to establish the link between mortality and sildenafil by reviewing the available literature in medical databases [8].

## Review

Methods

**Figure 1:**
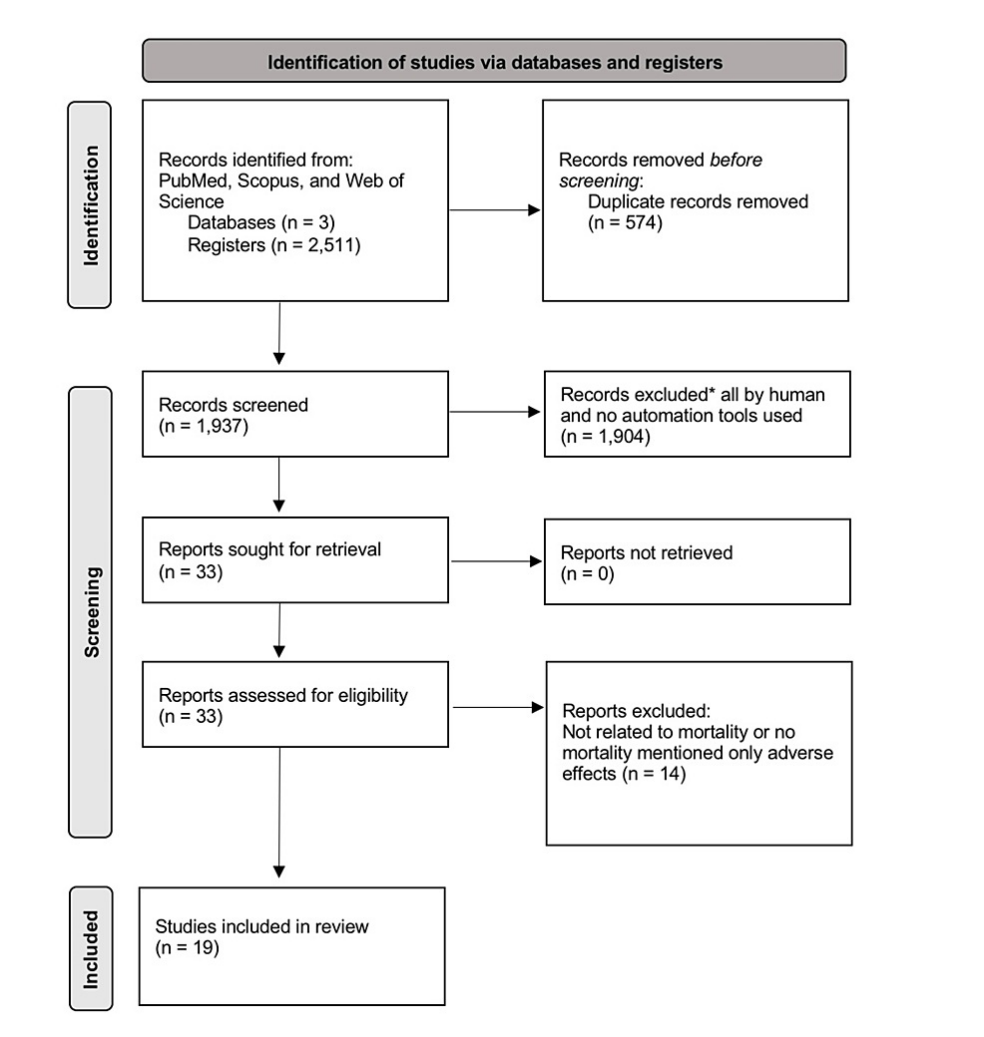
PRISMA flowchart PRISMA, Preferred Reporting Items for Systematic Reviews and Meta-Analyses

The systematic review was conducted in accordance with the Preferred Reporting Items for Systematic Reviews and Meta-Analyses (PRISMA) guidelines, as shown in Figure 1.

Screening of Articles

The inclusion criteria were papers published in English from 1998 onward that included males, females, and infants; all types of published research studies are included.

Exclusion criteria were any articles not conducted on humans and any articles not published in English.

Search Strategy

The screening of articles was carried out independently by seven reviewers. The aim was to acquire articles that pointed to sildenafil as a potential cause of death, thus suggesting the possible correlation between sildenafil and death. PubMed, Scopus, and Web of Science were used to search for publications. The following descriptors were used: sildenafil, Viagra, sildenafil citrate, PDE5 inhibitors, phosphodiesterase 5 inhibitors, death, postmortem, autopsy, and mortality. Boolean operators, such as “AND” and “OR,” were also used in the electronic search. The articles were randomly assigned to one of eight reviewers to extract data. The search was concluded on December 21, 2021.

Results and discussion

Sildenafil use has been rising steadily since its introduction in 1998, from being a drug primarily developed to treat heart problems to having a desirable yet unforeseen effect on the male genital system. Thus, it became one of the most commonly used drugs for the treatment of erectile dysfunction. Naturally, since every medication comes with side effects, which could ultimately result in death, studies have been conducted on sildenafil-associated mortality.

To collate the existing data on this topic, we read and reviewed the articles included in our systematic review to discuss the correlation between sildenafil and death. Case reports, reports to the FDA, and analyses were included in this discussion [8-26].

Sildenafil has been widely shown to be effective in treating erectile dysfunction. However, reports to the FDA have shown that its use was associated with 130 verified deaths and 112 unverified deaths just eight months after its introduction in March 1998. There were 1,473 major adverse events reported (including 522 deaths) after 13 months of availability [8].There are several advantages to initiating treatment with a low dose of sildenafil; the usual starting dose is 50 mg, which can be escalated to a maximum of 100 mg or titrated down to a minimum of 25 mg. These include identifying patients who are highly sensitive to sildenafil effects and cannot tolerate higher doses and decreasing the symptoms of flushing and dizziness that often affect compliance or adherence to treatment. This article proposes that sildenafil use is associated with different serious adverse effects, such as strokes, myocardial infarctions, and death, reported to the FDA in a period of eight months after sildenafil was introduced in the United States [18]. Moreover, some patients adversely react to the first dose of the agent, which suggests that sildenafil may provoke the first-dose reaction [15]. Therefore, extra caution is needed when starting treatment with sildenafil, a warning that was not stipulated by the guidelines at the time of its introduction to the U.S. market. An initial dose of 50 mg was prescribed for most men aged 18 to 65 years despite the differences in age, body weight, health status, and other medications used [10,15,18].

In 1998, when the drug was introduced and subjected to clinical trials around the world, the trials reported eight deaths. In addition, Israel reported six deaths to the FDA. At that time, the drug Viagra was counterfeited on a large scale in Lebanon and then smuggled and sold illegally in neighboring countries, leading to a prohibition of the drug in Palestine and a warning from authorities against the use of the drug [13].

Sildenafil has been used in the management of fetal growth restriction (FGR). A series of international randomized control studies aimed to assess the capability of sildenafil to improve the outcomes of FGR in gravid women. One of the studies included in this review mentioned that sildenafil’s harm was not significant in comparison to that of a placebo. However, a Dutch trial using sildenafil citrate to treat FGR was halted after the sudden deaths of 11 babies. The study reported that complications, including death, were more common in babies whose mothers were given sildenafil in comparison with mothers who were given a placebo. The suggested mechanism of toxicity is sudden cessation of the vasodilator agent, which is known as a type E (end-of-use or withdrawal) adverse drug reaction. This implies that sudden cessation of the drug carries an unpredictable risk of rapid deterioration [27].

The effect of sildenafil on infants was assessed in a retrospective study conducted to evaluate the mortality of patients younger than one year who received sildenafil. A total of 147 infants that did not have any congenital heart disease were included. Around 82% of them had severe pulmonary hypertension. The data showed a 29% mortality rate at discharge, and the mortality increased with increasing sildenafil dosage. This study is limited, however, as it did not have a control group, and external institutions conducted the investigations and thus these were not available for review [28].

Reviewing and analyzing the adverse effects of PDE5 inhibitors that have been reported to the FDA in a period of 10 years showed a total of 26,451 adverse events, of which death reports represent 8.3%. A total of 14,818 adverse events were reported with sildenafil used to treat erectile dysfunction and were associated with the highest number of death reports among the PDE5 inhibitors group, at 12.3% [19].

There were six cases in which the non-prescription use of PDE5 inhibitors may have contributed to the cause of death. This study suggests that the death of high-risk patients was induced by the use of PDE5 inhibitors. Autopsies revealed that four of the deceased were found to have a concentration of sildenafil between 0.032 and 0.087 μg/mL in the femoral blood (Table 1).

**Table 1:** Concentrations of sildenafil found in the femoral blood of four men during postmortem examinations taken from four cases described by Nagasawa et al. [9].

Case	Sildenafil concentration (μg/mL) in femoral blood
1	0.063
2	0.087
3	0.032
4	0.067

All the cases were associated with sexual activity; the first was a man in his 50s with uncontrolled hypertension. The second case was a man in his 40s with a history of fatal arrhythmia who died after having sex at a hotel. The third case was an elderly man in his 70s who died in a road traffic accident after spending a night away from home at an office party. He had a history of atrial fibrillation and combined use of PDE5 inhibitors. The fourth case was a man in his 80s complaining of dizziness and fever after having sex who then died after resting for a period. The contraindication to using PDE5 inhibitors in these patients was a history of cardiovascular disease in cases one and two. In case three, there was a use of combined PDE5 inhibitors, as revealed by toxicology screening, and the use of other drugs, amlodipine, telmisartan, and ethanol. In the last case, as found by autopsy, there was approximately 90% stenosis in the left anterior descending artery [9]. Studies showed that PDE5 inhibitors might contribute to death in patients with cardiovascular disease (after sexual intercourse) at low concentrations (0.040-0.105 μg/mL) [8,11,14,25,26].

In 2017, a case study reported the death of a 34-year-old male who was found unconscious near his residence. Security camera footage showed the deceased staggered; he collapsed as he was walking toward his vehicle. The deceased was not known to have any medical history, was not prescribed any medications, and was not taking over-the-counter medications. Autopsy findings were unremarkable, and there were no signs of trauma. Toxicological results showed no evidence of illicit drugs or alcohol. More testing revealed the presence of desmethyl carbodenafil, which is an unapproved sildenafil analog, at a concentration of 0.92 ± 0.13 mg/L in the man’s blood. In this case, the cause of death was listed as acute desmethyl carbodenafil toxicity [24].

Drug interaction with sildenafil was believed to cause death by predisposing a man in his mid-40s to sudden cardiac death. He was infected with HIV with arrhythmogenic right ventricular cardiomyopathy (ARVC). In regards to the co-administration of a drug that affects the metabolism of sildenafil, the patient was on a clarithromycin prescription for an itchy buccal rash. Clarithromycin is an antibiotic that inhibits CYP3A, which metabolizes sildenafil and increases sildenafil toxicity. Drug and toxin testing revealed a therapeutic concentration of sildenafil, suggesting that death occurred shortly after sildenafil ingestion. This drug interaction would have predisposed this patient with ARVC to sudden cardiac death [23]. A life-threatening adverse event was noted in patients with end-stage renal disease after they ingested 50 mg of sildenafil. This could be due to the higher concentration of the drug in the plasma of the patients with renal disease. Maximum plasma concentration (Cmax) was noted to be 70% to 80% higher in elderly patients or patients with renal impairment [29]. There was one case of a patient who was at the hospital and received medical care that restored his blood pressure. If this medical care had not been available, the patient could have died. This highlights that there is a life-threatening side effect for some patient groups and that caution is required before prescribing sildenafil to these groups [25].

Another report described an autopsy case for a man who died after a cerebral artery aneurysm followed by a subarachnoid hemorrhage after sexual intercourse, with toxicology reporting that sildenafil was found in the man’s blood. This report indicates that sildenafil affects vascular physiology through multiple different mechanisms. This case is an example of a rare association between sildenafil and a fatal pathological event, building on the existing knowledge of sildenafil and the involved pathophysiology of adverse events [21].

Another case described a 66-year-old male who collapsed during sexual intercourse with his spouse. The man was not known to have had cardiac problems nor did he take any medications regularly. For over a year, he took 50 to 100 mg of sildenafil orally 30 to 60 minutes before engaging in sexual intercourse. The wife also noted that the deceased had experienced mid-coitus chest pain and fatigue and that a few minutes after sexual intercourse, he collapsed. A postmortem examination was performed the following day. Pericardial tamponade was noted, with 200 mL of fluid in the pericardial sac alongside a 200-mg clot. The heart showed left ventricular hypertrophy. An aortic dissection involving both the ascending and descending part was also noted, the false lumen of which was filled with blood and blood clots. A toxicological examination that was performed with gas chromatography-mass spectrometry confirmed a high concentration of sildenafil (7.5 ng/mL). Acute hemopericardium resulting in cardiac tamponade was the main cause of death. The study concludes that the cause of death in this particular case is most likely the physiological changes associated with vigorous physical activity [22].

Another case reported an 80-year-old male whose putrefied corpse was found with a package containing 25 tablets of Viagra beside him. The family of the deceased said that he was complaining of chronic heart insufficiency and hypertension and that he had been taking beta blockers and diuretics regularly. An autopsy confirmed the presence of severe arteriosclerosis, pulmonary artery sclerosis, coronary artery sclerosis, heart enlargement, and several scars of the myocardium associated with previous myocardial infarctions. The study failed to demonstrate sildenafil as the cause of death, but it showed that the deceased did, in fact, take sildenafil prior to his death. Furthermore, sildenafil was contraindicated due to his previous myocardial infarction, severe arteriosclerosis, and hypertension [14]. Samples were also taken from a 60-year-old male found dead in his car. The man was found with sildenafil pills on him in circumstances that suggested he had recently taken part in sexual activity. Severe cerebral and coronary arteriosclerosis with pulmonary edema and signs of previous myocardial infarction were found during the autopsy. Toxicological examinations confirmed the presence of sildenafil in his body fluids and tissues. The identified cause of death was acute heart failure due to myocardiosclerosis [26].

In another case report, a 56-year-old male with intermediate cardiovascular risk was found dead at home with an empty box of Viagra (50 mg, 12 tablets) near the body. The autopsy examination found cardiac hypertrophy with coronary artery atherosclerosis and showed sildenafil in his blood at a concentration of 6.27 mg/mL [8]. In another case report, a 43-year-old man died in a hotel room during sexual intercourse, and a pillbox with several drugs, including 25 mg Viagra tablets and verapamil, was found in the room with two pills of Viagra missing. The man had been treated for cardiovascular disease and erectile dysfunction for several years. In the autopsy report of this case, there were multiple findings: heart enlargement, severe aortic arteriosclerosis, coronary sclerosis, and edematous lung. The toxicological report revealed that sildenafil was present in his blood, urine, gastric content, and hair, demonstrating chronic drug use; in addition, verapamil was found in the blood, measured above the therapeutic range. In this study, the results showed that, rather than causing a synergistic effect, sildenafil produces additive effects in reducing blood pressure when combined with verapamil, possibly indicating the cause of death in this case [11]. In another study, five autopsy cases related to sildenafil were assessed and showed similar findings. Four of the five cases died of cardiovascular disorders, while three of them tested positive for sildenafil in their blood [20]. The cardiovascular risk to sildenafil users has been studied since the drug’s release. However, it is unusual for cardiovascular disease to show symptoms that make patients aware of their condition prior to taking sildenafil without a medical checkup to determine their risk. Additionally, sexual intercourse increases cardiovascular risk by itself [20]. Table 2 depicts a summary of all the case reports reviewed in the present study and the concentration of sildenafil found.

**Table 2:** A summary of the papers that were reviewed for this study that examined deaths related to sildenafil. The circumstances of each death and the concentration of sildenafil found in the blood of the deceased during postmortem examinations are listed.

Title	Authors	Year of publication	Circumstances	Sildenafil concentration in blood
Association Between Sexual Activity-Related Death and Non-Prescription Use of Phosphodiesterase Type 5 Inhibitors	[9]	2021	(1) A man in his 50s collapsed after masturbating in front of a woman at a hotel and later died. (2) A man in his 40s returned home by car alone after having sex at a hotel and was found dead in the car the next day. (3) A man in his 70s died in a traffic accident on his way home. (4) After a man in his 80s had sex and bathed in a hotel, he complained of fever and dizziness and then died after a period of time.	0.063 (μg/mL) 0.087 (μg/mL) 0.032 (μg/mL) 0.067 (μg/mL)
A Fatal Hypotension by Sildenafil in an End-Stage Renal Disease Patient With Hypertension and Abnormal Pharmacokinetics of the Medicine	[25]	2009	A man in his 70s had severe kidney damage, loss of consciousness, and low blood pressure while using 50 mg as treatment.	0.44 in blood (μg/mL)
Last Performance With VIAGRA: Post-Mortem Identification of Sildenafil and Its Metabolites in Biological Specimens Including Hair Sample	[11]	2002	A man in 40s was treated for cardiovascular disorder and erectile dysfunction and died during sexual intercourse.	0.105 (μg/mL)
Postmortem Distribution of Sildenafil in Histological Material	[26]	2005	A man in his 60s was found dead in his car, and an autopsy showed severe coronary arteriosclerosis and myocardial infarction.	0.04 (μg/mL)
Fatal Overdosage With Sildenafil Citrate (Viagra): First Report and Review of the Literature	[8]	2002	A man in his 50s died of an acute 600 mg oral overdose.	6.27 (μg/mL)
A Case Report of Fatal Desmethyl Carbodenafil Toxicity	[24]	2017	A male in his 30s was found unresponsive at his residence. Toxicology testing revealed no alcohol or illicit drugs, more testing revealed the presence of desmethyl carbodenafil, which is an unapproved sildenafil analogue.	0.92±0.13 (mg/L)
Post-Coital Death in Chronic Sildenafil Abuser	[22]	2020	A man in his 60s collapsed during intercourse with his spouse. He had been taking sildenafil regularly for more than a year. The wife noted that the deceased experienced sudden chest pain and fatigue mid coitus and that he collapsed a few minutes after intercourse.	7.5 (ng/mL)

In summary, there are articles that have included the autopsy reports, and others have mentioned the comorbidities found in the deceased that could have been the primary cause of death. Other studies have halted their research due to the increased number of deaths in the study group. These studies have failed to establish a causality link between sildenafil use and mortality.

## Conclusions

Many articles emphasize the need to screen patients for cardiovascular risk before the prescription of sildenafil, but the over-the-counter availability of the drug in all pharmacies makes it hard to implement guidelines. As a countermeasure, sildenafil should be made a prescription drug that is prescribed after thorough evaluation of comorbidities, especially cardiovascular, and medications of patients such as nitrates. Moreover, patients who are prescribed drugs that are proven to have interactions with sildenafil should receive education regularly at their follow-up visits and offered alternatives to be used such as vacuum pump, alprostadil intraurethral pellet (suppository), intrapenile injections of alprostadil or papaverine, and surgery alternatives such as penile prostheses or penile revascularization. Other articles have emphasized the need to screen for sildenafil levels during an autopsy if the death was related to any sexual activity. To conclude, the literature available on this topic is deemed insufficient to provide enough data to establish a direct link of causality between sildenafil and mortality. Although some studies paint sildenafil as the culprit behind deaths such as these. Further studies, research, and complete autopsies with advice to check for sildenafil levels if a death is suspected due to sexual activity are needed to explain the unexpected deaths following the use of sildenafil.
